# Skin blood flow responses to locally applied acetylcholine in Caucasian and African descent individuals with and without cyclooxygenase inhibition

**DOI:** 10.1186/2046-7648-4-S1-A33

**Published:** 2015-09-14

**Authors:** Matthew Maley, James R House, Michael J Tipton, Clare M Eglin

**Affiliations:** 1 Extreme Environments Laboratory, Department of Sport and Exercise Science, University of Portsmouth, UK

## Introduction

Individuals of African descent (AFD) are more susceptible to non-freezing cold injury (NFCI) than Caucasians (CAU) [1]. This may be a consequence of lower skin blood flow during local cold exposure and subsequent rewarming in AFD [2], possibly due to a difference in endothelium function as acetylcholine (ACh)-induced vasodilatation is smaller in AFD than CAU on the non-glabrous finger and toe skin sites [3]. It is known that prostaglandins produced by the enzyme cyclooxygenase (COX) mediate part of the ACh-induced vasodilator response [4] however in hypertensive individuals, COX inhibition results in augmented vasodilatation in response to ACh [5] demonstrating that COX can also promote vasoconstriction. Whether COX products are involved in the attenuated vasodilator response to ACh in healthy AFD [3] is not known. Therefore, the aim of the present study was to investigate the contribution of COX in both CAU and AFD to local application of ACh in foot and finger skin sites which are susceptible to NFCI.

## Methods

12 AFD and 12 CAU male volunteers received local application of ACh (1 %). Participants consumed 150 mL of orange squash (placebo) prior to resting supine for 30 minutes in 23 °C. ACh was then applied using iontophoresis (six pulses of 25 μA followed by one pulse of 50 μA and one of 100 μA applied for 20 s with 60 s intervals) to the medial or lateral aspect of the dorsum of the foot and then the third or fourth finger. Following this, participants consumed 600 mg aspirin (COX inhibitor) dissolved in 150 mL of orange squash and remained resting for 30 minutes before undertaking the same iontophoresis protocol as before. The order of skin sites was balanced. Skin blood flow was measured by laser Doppler and maximum percentage change (max%Δ) and area under the curve (AUC) were calculated. Skin resistance was too high in some AFD and therefore iontophoresis was not possible in these individuals.

## Results

**Foot**: ACh elicited a greater vasodilatation in CAU than AFD following placebo (Median [interquartile], CAU n = 12, AFD n = 12, max%Δ: 943[490] % vs 81[370] %, P = 0.003; AUC: 4516[2601] vs 190[1329], P = 0.001) and aspirin (Median [interquartile], max%Δ: 775[784] % vs 50[148] %, P < 0.000; AUC: 3120[3170] vs 95[894], P = 0.002). Aspirin reduced the response to ACh in CAU only (AUC P = 0.031). **Finger**: ACh elicited a greater vasodilatation in CAU than AFD following placebo (Mean [SD], CAU n = 11, AFD n = 10, max%Δ: 301[76] % vs 160[139] %, P = 0.013; AUC: 1542[597] vs 539[660], P = 0.002) and aspirin (Median [interquartile], CAU n = 11, AFD n = 8, max%Δ: 287[162] % vs 53[88] %, P = 0.001; Mean [SD], AUC: 1255[872] vs 35[218] P = 0.001). Aspirin showed a tendency to reduce the response to ACh in AFD (max%Δ, P = 0.064; AUC, P = 0.053) but not CAU.

## Discussion

CAU have a greater endothelial reactivity than AFD in both foot and finger skin sites irrespective of whether COX was inhibited or not. In response to local ACh application, it appears that the role of prostaglandins in AFD is minimal in the foot and finger. In CAU, prostaglandins appear to play a role in dilatation in the foot only.

## Conclusion

The present study demonstrates that the lower endothelial reactivity in AFD, as evidenced by a reduced vasodilation to ACh, is not due to alterations in the COX pathway.
